# t(9;19)(q22;p13) in Acute Myelomonocytic Leukemia

**DOI:** 10.4274/tjh.2017.0368

**Published:** 2018-03-06

**Authors:** Moeinadin Safavi, Akbar Safaei, Marzieh Hosseini

**Affiliations:** 1 Tehran University Faculty of Medicine, Department of Pathology, Molecular Pathology and Cytogenetic Ward, Tehran, Iran; 2 Shiraz University of Faculty of Medicine, Department of Pathology, Molecular Pathology and Cytogenetic Ward, Shiraz, Iran

**Keywords:** Acute myeloid leukemia, Cytogenetic, Monocytic differentiation

## To the Editor,

Chromosomal aberrations play a role in the leukemogenesis of acute myeloid leukemia. Some chromosomal abnormalities such as t(8;21), t(15;17), and inv(16) are frequently observed, but hundreds of uncommon chromosomal translocations also exist and their significance remains to be clarified [1]. Here we introduce a case of acute myeloid leukemia with a very rare translocation and explain its morphologic and immunophenotyping findings.

**Figure 1:**
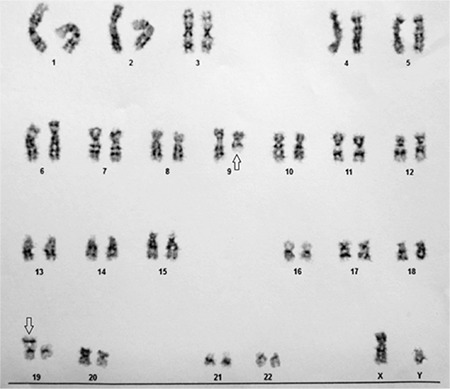
Bone marrow karyotype study revealed t(9;19)(q22;p13).

The patient was a 50-year-old man with malaise and weakness. Paraclinical evaluation revealed leukocytosis along with anemia and thrombocytopenia (white blood cells: 24,000/µL, hemoglobin: 7.4 g/dL, platelets: 30,000/µL). Peripheral blood smear exhibited atypical blastoid cells. Subsequently the patient underwent bone marrow aspiration, which showed 80% blasts of myeloid and monocytic type with prominent cytoplasmic vacuolization. Immunophenotyping by flow cytometry revealed positive reactions for CD117, HLA-DR, MPO, and CD64. Morphologic findings and immunophenotyping were compatible with acute myelomonocytic leukemia. Bone marrow cytogenetic study showed t(9;19)(q22;p13) (Figure 1). Reverse transcriptase PCR was performed for t(8;21) (*AML1-ETO* fusion gene) and inv(16) *(CBFB-MYH11* fusion gene), which was negative for both of them. *FLT3* duplication and *D835* mutation were also negative. Subsequently, the patient underwent a 7+3 chemotherapy regimen with cytarabine continuous infusion (300 mg, IV) over 24 h on days 1 to 7 and daunorubicin (115 mg, IV bolus) on days 1 to 3. Although remission was achieved after induction therapy (3% blasts in bone marrow 4 weeks after chemotherapy), unfortunately the patient contracted sepsis due to neutropenia and died 1.5 months after treatment initiation.

Acute myeloid leukemia with prominent monocytic lineage involvement (M4-M5) is usually associated with determined recurrent cytogenetic aberrations like inv(16), t(v;11) (*MLL* gene rearrangement), and t(8;16). According to a literature review, t(9;19)(q22;p13) has been reported previously only twice. The first case was a 57-year-old man with acute myelomonocytic leukemia and concomitant inv(16). Exact morphologic and immunophenotyping characteristics of this case were not determined [2]. The second case was a 13-year-old boy with acute myeloid leukemia (M0) who developed multiple clonal abnormalities during his treatment course [3]. The present case is the first patient with acute myelomonocytic leukemia with t(9;19)(q22;p13) as the sole chromosomal abnormality. This cytogenetic finding and its associated morphologic and immunophenotyping characteristics are noteworthy and merit attention.
